# Recent Progress in Proteins-Based Micelles as Drug Delivery Carriers

**DOI:** 10.3390/polym15040836

**Published:** 2023-02-08

**Authors:** Aleena Mustafai, Muhammad Zubair, Ajaz Hussain, Aman Ullah

**Affiliations:** 1 Institute of Chemical Sciences, Bahauddin Zakariya University, Multan 60800, Pakistan; 2 Department of Agricultural, Food and Nutritional Science, Lab# 540, South Academic Building, University of Alberta, Edmonton, AB T6G 2P5, Canada

**Keywords:** micelles, proteins, drug delivery, biomedical, biocompatible

## Abstract

Proteins-derived polymeric micelles have gained attention and revolutionized the biomedical field. Proteins are considered a favorable choice for developing micelles because of their biocompatibility, harmlessness, greater blood circulation and solubilization of poorly soluble drugs. They exhibit great potential in drug delivery systems as capable of controlled loading, distribution and function of loaded agents to the targeted sites within the body. Protein micelles successfully cross biological barriers and can be incorporated into various formulation designs employed in biomedical applications. This review emphasizes the recent advances of protein-based polymeric micelles for drug delivery to targeted sites of various diseases. Most studied protein-based micelles such as soy, gelatin, casein and collagen are discussed in detail, and their applications are highlighted. Finally, the future perspectives and forthcoming challenges for protein-based polymeric micelles have been reviewed with anticipated further advances.

## 1. Introduction

Presently, various drug delivery systems such as micelles, nanogel, nanocrystals, nanotubes and nanocapsules are used to deliver drugs and therapeutically active molecules. These nano-ranged drug delivery systems are preferred over the use of conventional drugs because they have minimized the limitations of drugs such as toxicity, poor biostability, poor solubility and multidrug resistance [1,2]. The most familiar case in this perspective is the dissolution of water-insoluble drugs as facilitated by the hydrophobic cores of micelles. As a result, the drug can be loaded for delivery at the targeted sites, which reduces the drug loss and harmful effects along with an increase in drug bioavailability at the required zone [3,4,5].

The polymeric micelles are considered good vehicles for drug delivery because of their stability, nano size, surface characteristics and enhanced permeability and retention (EPR) effect [6]. The self-assembly of amphiphilic block co-polymers allows the polymeric micelles into a core–shell structure and drugs are loaded in the core of polymeric micelles. The size of polymeric micelles ranges from 10 to 100 nm [7,8]. The hydrophobic core of polymeric micelles is mainly composed of polyesters, poly (L-amino acids) and polycaprolactone. In contrast, the hydrophilic shell of polymeric micelles is primarily composed of polyethylene glycol (PEG) [9].

In a dilute solution, amphiphilic molecules exist separately in the form of surfactants. At higher concentrations, these unimers undergo self-aggregation to form a core–shell structure called micelles. The minimum concentration of polymers at which the formation of micelles occur is called critical micelle concentration (CMC) [10]. CMC is an essential parameter for determining the thermodynamic stability of micelles. The concentration of polymers above CMC is important for micelles stability while the concentration of polymers below CMC causes the disassembly of micelles into their unimers. In addition to thermodynamic stability, kinetic stability is also an important parameter [11,12,13]. Micelles composed of low molecular weight surfactants dissociate in microseconds while polymeric micelles preserve for longer due to their high molecular weight and low CMC. High kinetic stability and low toxicity of polymeric micelles over low molecular weight surfactant-based micelles make them suitable for their preferred use in the drug delivery [14,15].

Biocompatible ligands modify polymeric micelles for active targeted drug delivery. Commonly used ligands are antibodies, sugar moieties, peptides and proteins [16,17,18]. The degradation of micelles releases drugs at the targeted site in response to the stimuli such as temperature, pH, and unregulated enzymes. External stimuli such as light, ultrasound and magnetic field can also cause drug release from micelles [6,19].

Biodegradable and non-biodegradable nanomaterials could be used for drug delivery, but biodegradable materials are preferred due to their better feasibility and applicability [15]. Biopolymer-based micelles are highly valuable because they show the characteristics of both micelles and biopolymer [13,20]. These micelles are widely used as drug carriers because their core solubilizes the large number of hydrophobic and hydrophilic substances, and the corona (external surface) protects it from the reticuloendothelial system.

Protein-based systems are preferred over the others for drug delivery to a specific position inside the human body because of many advantages such as proteins amphiphilic nature (water soluble and water insoluble), non-ecotoxicity and easy modification for targeted drug delivery applications [21]. Protein-based drug delivery systems are responsible for sustained and targeted drug delivery at the tumor site and are used in cancer therapy, pulmonary therapy and vaccines [22].

## 2. Proteins in Drug Delivery

- Micelles based on natural biopolymers are ideal drug carriers because of good compatibility, biodegradability, non-toxicity, prolonged blood circulation time and non-immunogenicity, and they can release drugs at the desired site.
- Protein-based drug delivery systems are used for cancer therapy, tumor therapy, drug delivery to lungs and vaccination due to their non-antigenic properties. Drug delivery systems based on proteins have many compensations such as stability, biocompatibility, biodegradability and the comfort of controlling particle size.
- Protein-based drug delivery systems can undergo surface modification due to the presence of various functional groups such as carboxyl, amino and hydroxyl groups, and can bind significant amounts of drugs through hydrophobic interactions, covalent bonds and electrostatic interactions. Due to the presence of different functional groups, proteins deliver drugs at the specific site inside the body [13].
- Because of their small size, protein-based nano micelles are highly suitable for intravenous drug delivery. They can efficiently deliver drugs in blood stream [23].
- Proteins are biodegradable and converted into non-toxic substances easily assimilated by the body; protein-based drug carriers are safer. Preparation of protein nanoparticles and drug encapsulation in protein-based micelles require mild conditions without toxic chemicals and solvents [24].
- Proteins are biocompatible, on absorbing water and creating space repulsion, they stabilize nanoparticles and reduce their recognition by the immune system of the body [25,26].

## 3. Forms of Drug Delivery Micelles

The micelles may adopt a variety of arrangements of the molecules involved in the formation of micelles. These arrangements may lead to the formation of either a hydrophilic or hydrophobic type of internal core or the corona (external surface of micelles). Similarly, the number of molecules involved in the micellization process may affect the micelles’ form. Moreover, the nature of molecules aggregating to form micelles may also affect the form adopted by micelles. Various micelles have been developed for drug delivery systems depending on the nature of applications. So far, the following are the most used micelles, as discussed below.

### 3.1. Regular Micelles

Regular micelles are formed by hydrophobic interactions [27,28]. Regular micelles are obtained when amphiphilic copolymers are self-assembled in an aqueous medium so that the hydrophilic region is oriented outside, and the hydrophobic portion is oriented inside. Regular micelles also deliver poorly soluble drugs by enhancing solubility in an aqueous medium—for example, polyethylene glycol-polylactic acid, polyethylene oxide-polypropylene oxide, polyethylene glycol-polylactic-co-glycolic acid. Regular micelles are also being used in the suppression of excited state reactions of different dye molecules. Auramine O photophysics were studied by adsorbing it on regular micelles’ surface and in bulk water. The results showed that the reaction rate at the interface of water and regular micelles was slower than that in bulk water [27].

### 3.2. Reversed Micelles

Reverse micelles are formed when amphiphilic copolymers self-assembled in a non-aqueous medium, the hydrophobic region orient outside and the hydrophilic portion orient inside. These are used to deliver hydrophilic drugs and proteins in a non-aqueous medium. For example, phosphazene micelles in chloroform, polycaprolactone-poly (2-vinyl pyridine) micelles in the oleic acid [28]. Various amphiphilic molecules are being used for the formation of reversed micelles. Anionic sodium 1,4-bis-2-ethylhexylsulfosuccinate (AOT)-based reversed micelles can solubilize greater number of hydrophilic substances depending on temperature and surrounding hydrophobic medium. Cationic amphiphilic molecules benzyl-n-hexadecyldimethylammoniumchloride (BDGC)-based reversed micelles are synthesized in aromatic solvents only. The combination of AOT and BDGC generates AOT-BHD-based reversed micelles that form unilamellar vesicles in water and the solubility of this new moiety is due to the cationic portion [29].

### 3.3. Unimolecular Micelles

Unimolecular micelles are formed when many hydrophobic and hydrophilic regions are present in one molecule, and that one molecule is self-assembled by hydrophobic interaction to form micelles. For example, core (Laur) polyethylene glycol micelles in an aqueous medium. Their structural stability is maintained under extreme environmental conditions such as dilution, temperature change and pH change [28]. Unimolecular micelles are used to deliver drugs by physically encapsulation drugs or by forming covalent bond with drugs. Yao et al. designed unimolecular micelles based on PAMAM-g-poly[3-dimethyl(methacryloyloxyethyl) ammonium propanesulfonate] (PAMAM3.0-g-PDMAPS) for the encapsulation of DOX. PAMAM3.0 is the hydrophobic core and PDMAPS is the hydrophilic shell which stabilizes micelles and prevents the adsorption of non-specific proteins on unimolecular micelles. Unimolecular micelles are also used as carriers in catalysis and as templates for inorganic nanoparticle formation [30].

### 3.4. Mixed Micelles

Mixed micelles are obtained by blending different polymers. They are generated to increase the thermodynamic stability of micelles and to produce small micelles with enhanced drug-loading capacity compared to micelles consisting of an individual component. For instance, 1,2-stearoyl-sn-glycerol-3-phosphoethanolamine-N-methoxy poly (ethylene glycol) and poly (ethylene glycol)-b-poly(E-caprolactone) (PEG5000-b-PCLx) serve the purpose of mixed micelles [3]. F127/TPGS-based mixed micelles were used for the encapsulation of DOX and exhibited 3.9- and 12.2-folds greater toxicity for MCF-7 breast cancer cells and THP-1 leukemia cell lines. Lin et al. investigated the pH-influenced drug-releasing behavior of polyGlut-b-PPO-b-polyGlut and PEG-b-PPO-based mixed micelles. They noted that the drug release rate increased when the solution’s pH decreased from 7 to 4 [31].

### 3.5. Polyion Complex Micelles (PICMs)

These are formed by the interaction between oppositely charged polymers. These micelles are also known as complex coacervate core micelles, block ionomer micelles and interpolyelectrolyte micelles. Electrostatic interactions develop between two oppositely charged polymers when they are added to the aqueous medium. As a result, polyionic micelles are obtained as polyion complex micelles (PICMs). Protein-based PICM is prepared by condensation of a block copolymer with charged protein block or neutral protein block. Condensation of negatively charged protein with positively charged block polymer and condensation of positively charged protein with negatively charged protein resulted in protein-based PICMs. If PICM is formed by the condensation of neutral polymer with charged protein block, then the neutral block stabilizes the charged block of protein [32].

## 4. Methods for Micelles Preparation

The methods employed to prepare micelles depend upon the solubility or film forming ability of drugs and the polymer molecules involved in micelles formation. However, certain techniques, such as dialysis, evaporation of solvents and microphase separation, are also used for micellization. The involvement of oils also involves the formation of emulsions leading to micellization. In this regard, different methods for micelles preparation have been reported in the literature. The recently used methods for preparation of drug delivery micelles are discussed below.

### 4.1. Direct Dissolution

In the direct dissolution method, copolymer and drugs are dissolved in an aqueous solvent [33]. It is the simplest method of preparing micelles using highly water-soluble polymers. Micelles are obtained by self-assembly polymers when polymer concentration is more than critical micelle concentration (CMC) [34]. The direct dissolution method has been used for the preparation of polylactide/poly(ethylene glycol) (PLA/PEG)-based micelles easily for the encapsulation of paclitaxel without the use of toxic organic solvent. The results showed that the micelles prepared by this method exhibited greater drug encapsulation efficiency and the solubility of paclitaxel in water increased 1000-fold [35].

### 4.2. Dialysis

This method involves the formation of a solution of polymer and drug in water-miscible organic solvents such as dimethyl formamide, methanol, ethanol, tetrahydrofuran and acetone, followed by dialysis with water for several hours to remove all the water-miscible organic solvents which result in the formation of micelles. The dialysis preparation method’s drawback is that it requires more time and generates wastewater [20,28,34]. Curcumin loaded poly(lactide-co-glucolide)-b-poly(ethylene glycol)-b-poly(lactide-co-glycolide) (PLGA-PEG-PLGA) copolymer has been developed by dialysis method. Curcumin and PLGA-PEG-PLGA copolymer were dissolved in acetone and treated with ultrasonics. The mixture was dialyzed using a dialysis membrane against water. Then, this mixture was filtered to remove undissolved curcumin [36].

### 4.3. Oil in Water Emulsion Evaporation

In this method, hydrophobic drugs are allowed to dissolve in water-immiscible and volatile organic solvents such as chloroform, dichloromethane, and ethyl acetate, and are added to the aqueous polymer solution. As a result, nano-emulsions are obtained and the volatile organic solvent is then allowed to evaporate to obtain drug carrying micelles [20,28]. Indomethacin (IMC) was encapsulated into poly(ethylene oxide)-poly(β-benzyl L-aspartate) (PEO-PBLA) micelles by using the oil in water emulsion method. For this purpose, IMC was dissolved in chloroform and PEO-PBLA micelles were dissolved in water. The solution of IMC was added dropwise into the solution of PEO-PBLA micelles with continuous stirring in open air to remove chloroform. The solution was filtered by ultrafiltration to obtain IMC loaded micelles. the amount of IMC entrapped in PEO-PBLA micelles was 22.1%w/w [37].

### 4.4. Co-Solvent Evaporation

In this technique, drugs are allowed to dissolve in solvents such as methanol, and the polymer is dissolved in distilled water. These two solutions are mixed to give clear solutions, which is allowed to evaporate in rotatory evaporator for several hours at specific temperatures and pressure to obtain drug-loaded micelles [28]. This method was used for the encapsulation of cyclosporine A drugs into methoxy poly(ethylene oxide)-b-poly(ε-caprolactone) (MePEO-b-PCL)-based copolymers. In this method, solution of MePEO-b-PCL was made in organic solvent (THF, acetone or acetonitrile). The solution of micelles was added dropwise into water with vigorous stirring and vacuum was applied to eliminate organic solvent. After this, the solution of drugs in organic solvent was added for encapsulation of drugs into micelles. In the end, the solution was centrifuged to remove precipitates of cyclosporine A [38].

### 4.5. Microphase Separation

Copolymer and hydrophobic drugs are dissolved in a volatile organic solvent such as tetrahydrofuran and then added into an aqueous phase dropwise with continuous stirring to remove the volatile organic solvent. As a result, drug-encapsulated polymeric micelles are obtained [39]. Poly(lactic acid)-polyurethane (PULA) based micelles synthesized by this method showed enhanced biocompatibility, drug storage and drug releasing facility. For the preparation of PULA micelles, a solution of PULA and drug was made in organic solvent (THF) and added dropwise in water with continuous stirring. The organic solvent was removed under reduced pressure to obtain drug-loaded micelles [40].

### 4.6. Thin Film Hydration

Copolymer and hydrophobic drugs are dissolved in the organic solvent. After the evaporation of solvent by the rotary evaporator, a thin film is obtained to which an aqueous phase is added for hydration and drug-loaded micelles are formed [13]. Thin film hydration is a simple and practical method used to encapsulate DOX into disulfide linked polyethylene glycol 5000-lysin-di-tocopherol succinate (P_5k_SSLV) for the development of DOX-loaded P_5k_SSLV micelles. P_5k_SSLV copolymer, DOX and triethylamine were added to organic solvent and mixed. The solution undergoes vacuum evaporation of remove organic solvent and is dried in nitrogen environment to form a lipid membrane containing the drug. Buffer solution was added in the drug-loaded lipid membrane, heated, stirred, centrifuged and filtered to obtain nanomicelles [41].

## 5. Types of Proteins Used as Micelles for Drug Delivery

Various proteins have been studied in drug delivery systems. Soy, collagen, gelatin, casein and albumin are the mainly used proteins-derived materials for drug delivery applications, as shown in Table 1. In this section, the micelles obtained from each protein have been discussed.

### 5.1. Gelatin

Gelatin is a denatured collagen found in organisms’ skin, tissues and connective tissues. It is a natural water-soluble polymer with several medical applications due to its biocompatibility and non-toxic nature. Gelatin-based drug delivery systems are considered responsible for the sustained release of hydrophobic drugs and proteins [42]. Gelatin decomposes rapidly and has low mechanical stability, so it has to be linked with crosslinkers such as GA (glutaraldehyde) to lower decomposition—higher crosslinking density results in a low decomposition rate [43]. Gelatin has a specific repetitive sequence of amino acids containing glycine at every third position Ala-Gly-Pro-Arg-Gly-Glu-Hyp-Gly-Pro-, that is responsible for the improved biological activity of gelatin [44].

Gelatin modification with PEG (polyethylene glycol), known as PEGylation, enhances the drug carrier’s circulating time by reducing immunogenicity because the hydrophilicity of PEG does not allow protein adsorption on the surface of drug carriers. PEG-modified gelatin is involved in the delivery of noscapine (an alkaloid) in human carcinoma associated with non-somal lung cells. The thiolation of PEG-modified gelatin improves the biostability of nanoparticles by forming disulfide bonds. Ethylene diamine, polyethylenimine and spermine are used to “cationize” the gelatin involved in the delivery of small interfering RNA (siRNA) to preclude the spread of peritoneal fibrosis in mice by suppressing type-III collagen. The association of type plasminogen activator (tPA) with positively polarized PEGylated gelatin suppresses bleeding complications caused by tissue tPA. Lactic acid modified gelatin delivers a hydrophobic hydrolipidermic drug, simvastatin. The grafting of gelatin with hexanoyl anhydride entraps the hydrophobic anticancer drug camptothecin. Oleic acid modified-gelatin is specifically for gastric and intestinal drug delivery. The introduction of epidermal growth factor receptor (EGFR) recognition sequence on gelatin is used for gene delivery studies in the cancer cells of the pancreas [45].

Gemcitabine (GEM) encapsulated-gelatin nanoparticles were developed by Amit Singh et al. for the treatment of pancreatic cancer. Gelatin NPs were coated with polyethylene glycol (PEG) for targeted delivery and enhanced drug circulation time. To enhance therapeutic efficiency and to reduce the side effects of therapeutic drugs doxorubicin (DOX) and betanin, PEGylated gelatin NPs were developed by Sajed Amjadi et al., which exhibited pH-responsive controlled release of DOX and betanin at the tumor site. Uyen Vy Vo et al. designed poly (ethylene glycol) methyl ether (mPEG) functionalized gelatin porous nanosilica (PNS) nanoparticles for loading DOX to evaluate its oral delivery potential. The obtained nanoparticles exhibited pH-responsive sustain release of DOX at the targeted site [46].

Lu et al. prepared paclitaxel-loaded gelatin nanoparticles for the treatment of intravesical bladder cancer. Gelatin-based drug delivery systems protect the dilution of paclitaxel by urine production and prevent therapeutic failure. These nanoparticles are responsible for the sustained release of drug that would prevent the change in the concentration of paclitaxel with the volume of urine. Wang et al. developed gelatin nanoparticles modified with 3-carboxyphenylboronic acid (3-CPBA) to encapsulate DOX. 3-CPBA ligands specifically recognize increased level of sialic acid due to its overexpression by tumor cells. DOX-loaded 3-CPBA modified gelatin nanoparticles exhibited improved antitumor activity and tumor accumulation compared to free drugs. Hu et al. prepared a gelatin-dendritic poly-L-lysine (DGL)-based system for drug delivery. DOX conjugated with DGL and encapsulated into gelatin nanoparticles has been reported. The hydrolysis of gelatin by metalloproteinases (MMP-2) in the tumor environment and release of DOX/DGL enabled the permeation of DOX into the core tumor. Karthikeyan et al. employed resveratrol after loading in gelatin nanoparticles to successfully treat lung cancer. These nanoparticles target NCI-H460 lung cancer cells and exhibit improved antitumor activity. A combination of gelatin nanoparticles and iron oxide suspension led to the generation of magnetic gelatin nanoparticles to encapsulate gemcitabine. pH dependent release of gemcitabine from nanocarriers for the treatment of pancreatic cancer [47].

Gelatin nanoparticles were modified with hyaluronic acid (HA) to encapsulate epigallocatechin gallate. HA improved the adhesion property of gelatin nanoparticles with mucus and enhanced the survival time of drugs involved in the treatment of dry eye in rabbits [48]. HA-modified gelatin nanoparticles containing carboxymethyl chitosan (CC) were developed to encapsulate curcumin in the treatment of inflammatory bowel disease. HA acts as an anionic carrier that prevents or repair the inflamed intestine. The resultant nanocarriers also have potential of effective drug delivery in the treatment of colitis. CC enhanced the therapeutic effect of curcumin in the colon site and exhibited improved mucosal adsorption in the colon [49].

Piao and Chen developed a self-assembled graphene oxide-gelatin nanocomposite that worked as a pH-responsive drug delivery system. Alemdar et al. showed that bone ash-conjugated gelatin/alginate/hyaluronic acid composites could be used for pH-responsive controlled drug release. Ooi et al. displayed that cellulose-reinforced gelatin materials could be used as pH-responsive controlled release drug delivery system [50]. D-glucose stabilized gelatin/collagen matrix was used to deliver calendula afficinalis powder and oil that significantly enhanced anticancer activity towards human breast cancer cells (MCF7 cells) and human hepatoma cells (SKHepi cells) [51].

Redox and MMP-2-responsive gelatin nanoparticles were developed for the delivery of paclitaxel (PTX) by utilizing glutathion (GSH) and MMP-2 that is over-expressed around some tumor tissues. The targeted ligand bovine serum albumin (BSA) was further employed for active targeted delivery of the drug. PTX-SS-COOH was grafted onto sulfhydryl modified gelatin (Gel-SH) through an amide bond to produce Gel-SS-PTX amphiphilic polymer conjugated with BSA through electrostatic force and hydrogen bonding to produce BSA/Gel-SS-PTX/PTX-SS-COOH nanoparticles. These nanoparticles reached the targeted site through EPR effect and were triggered by MMP-2 to release the PTX-SS-COOH first, then conjugated PTX was released at tumor site [52].

Poly(L-lactide) (PLLA) is a synthetic polyester used in the field of tissue engineering and controlled drug release due to its biocompatibility and non-toxicity. Grafting of gelatin with PLLA takes place in N-hydroxy succinimide (NHS) and 1-ethyl-3-(3-dimethylaminopropyl) carbodiimide hydrochloride (EDC), which serve as coupling agents. Gel-g-PLLA micelles were used as carriers for the anticancer drug Paclitaxel and showed sustained drug release. The encapsulating efficiency of micelles was 55% and showed the burst release of 70% drug within 24 h [53].

Triple-negative breast cancer (TNBC) is a malignant tumor that does not express receptors on its surface. Gelatin-oleylamine conjugate (GOC) is self-assembled in an aqueous medium to form micelles. Gelatin is conjugated with oleylamine in the presence of cross linker genipin and is involved in treating rare cancer cells such as TNBC cells. GOC nanocarriers encapsulate hydrophobic and less orally available antioxidant drug Catechin that treats TNBC. The TNBC cells MDA-MB-231 are bound to GOC nanocarriers and exhibit high cytotoxicity to cancer cells [54].

### 5.2. Collagen

Collagen is found in the human body’s extracellular matrix (ECM) that regulates cellular behaviors and tissue functions. Collagen contains the Arg-Gly-Asp sequence and is responsible for cell adhesion, proliferation and differentiation. Collagen-based drug delivery systems have several biomedical uses such as wound healing, drug delivery and tissue engineering [55]. Collagen-based nanoparticles are extensively studied for tissue engineering because they are suitable for nerve tissue regeneration due to their mechanical and physical properties. NeuraGen and Neuromaix are collagen-based formulations clinically approved in neural tissue engineering due to their effective peripheral nerve regeneration property [56]. Collagen transmits bioactive molecules and cellular components involved in myocardial regeneration and repair. The conjugation of collagen with other substances promotes the regeneration of peripheral nerves. For example, in dogs and rats, collagen-based agents are involved in sciatic nerve regeneration [57].

Collagen modified nanoparticles specifically bound to cartilage help to recover the structure and function of cartilage in the extra cellular matrix (ECM) by facilitating targeted drug release. Collagen-hybridized peptides have been developed that specifically bound to a denatured collagen strand and form a triple helical structure. Collagen type II prevents joint destruction, chondrogenic hypertrophy and pain in the treatment of osteoarthritis [58]. Collagen and hydroxyapatite (Col/HA) conjugated with bisphosphonate (BP) derived liposomes has excellent bone repairing effect. BP covalently binds to the liposome’s hydrophobic head, forming a hydrophilic tail by self-assembly in an aqueous medium. Mixing collagen with BP-liposome provides mechanical stability and prolongs drug release due to electrostatic interactions between BP-liposome and HA [59].

Collagen-based drug delivery systems have a high potential to regenerate uterine horns, and crosslinked collagen-HA matrix containing antibodies have high healing efficiency. Collagen protects and transmits proteins by binding the proteins with their interacting sites. A bi-affinity delivery system is developed when basic fibroblast growth factor (bFGF) is encapsulated by collagen to form a positively charged complex and then bounded with negatively charged heparin. This bi-affinity system protects bFGF from degradation in response to external stimuli. A collagen-pDNA delivery system was obtained by binding polylysine (PLL) with collagen involved in the sustained release of pDNA. A siRNA delivery system is obtained by loading nanostructured lipid carriers (NLC)/siRNA complex into collagen. NLC-loaded collagen exhibited a long-time release of siRNA and down regulated the expression of extracellular signal regulated kinase 1 (ERK-1) [60].

Zhong Luo et al. developed collagen-capped mesoporous silica nanoparticles for the targeted delivery of cancer drugs. The collagen-capped mesoporous silica nanoparticles are excellent chemotherapeutic drug carriers due to their high biocompatibility, excellent cellular uptake and targeted drug delivery compared to free drugs [46]. The obtained nanoparticles exhibit redox-responsive aspects of drug release.

Guo and coworkers studied ibandronate-loaded collagen that exhibited improved bone healing properties in osteoporotic rats. This drug delivery system showed enhanced cell adhesion, migration and callus formation compared to unloaded drugs. Maehara and coworkers develops FGF-2 (fibroblast growth factor) loaded hydroxyapatite modified collagen that exhibited improved bone repairing properties. Komaki and coworkers prepared tricalcium and collagen-based drug delivery systems for the delivery of FGF-2. The same system has also been used to deliver PDGF (platelet derived growth factor) [61].

**Table 1:** Proteins derived materials as drug delivery carrier.

Proteins	Materials	Methods of Generation	Biomedical Activities	Drugs	Ref.
Gliadin	Nanoparticles	Desolvation, Electron spray technique	High affinity for upper gastrointestinal tract, prolong residence time and induce cancer cell death.	Amoxicillin	[43]
Albumin	Nanoparticles	Desolvation,	Reduce drug leakage in gastric fluid.	Curcumin	[62]
	Nanoparticles	Thermal gelation	Increase cellular uptake and toxicity for A549 cells	Dimethyl curcumin	[63]
	Nanoparticles	Water-oil single emulsion.	Help to indicate cellular uptake and internal trafficking in macrophage cells.	Cefamandole nafate	[64]
	Nanospheres	Self-assembly	Enhance cellular uptake and nuclear accumulation.	Doxorubicin	[65]
	Micelles	Self-assembly	High loading efficiency for anticancer drug with enhanced cytotoxicity and cellular uptake.	Camptothecin	[66]
Zein	Nanoparticles,	Desolvation,	Enhanced solubility of drug in intestinal fluid without reducing its efficiency.	Glibenclamide	[67]
	Nanoparticles	Desolvation	Reduced astrogliosis, improve cognition and memory impairment. Increase bioavailability and antioxidant activity.	Quercetin	[68]
	Micelles	Self-assembly	Promising carriers of drug in cancer therapy. Decrease protein adsorption on micelles and uptake of micelles by macrophages.	Nile red	[69]
Casein	Micelles	Coacervation	Improved oral bioavailability	Quercetin	[70]
Gelatin	Micelles	Self-assembly	High toxicity for MDA-MB-231 cancer cells and used in the treatment of breast cancer.	Catechin	[71]
Elastin	Micelles	Self-assembly,	Thermal and pH sensitive drug release.	Geldanacym,	[54]
	Micelles	Self-assembly	Exhibit high antitumor activity for mice breast cancer cells. Involve in the treatment of Sjogren syndrome.	Rapamycin	[72]
Keratin	Micelles,	Cross-linking,	Exhibit dual (reduction and pH) responsive antitumor activity against HepG2 cells.	Doxorubicin	[73]
	Micelles	Self-assembly	High toxicity against A549 cells and exhibited triple (enzyme, glutathione and pH) responsiveness.	............	[74]

### 5.3. Casein

Casein is a phosphoprotein present abundantly in milk in aggregated form (micelles). Casein proteins are classified into different types (alpha, beta, k-casein) based on the number of amino acids, phosphorous and carbohydrate content. Casein proteins have both hydrophobic and hydrophilic portions in their structure [44]. Casein is used in drug delivery systems due to its stability, surface activity, self-assembly, emulsification, gel forming ability and binding of various molecules. It is used as a tablet coating material due to its high tensile strength. It acts as a carrier of anticancer drugs, and beta-casein reduces the growth of gastric cancer cells [26]. Sometimes, casein proteins cause allergic reactions, and there is a possibility of immunogenicity that limits its use in drug delivery [44].

Graft copolymers that are based on casein and N-isopropylacrylamide self-assembled to form micelles that have been used as drug carriers for DOX by the development of ionic interactions. These micelles are effective against breast cancer cells MDA231 and showed enzymatic, thermal and pH responsive drug release at the tumor site. Trypsin is overexpressed by some tumor cells that are detected by casein-N-isopropylacrylamide micelles. These nanocarriers showed very low toxicity and bioaccumulation due to their enzyme active degradation property [75].

Alginate has been effectively used in drug delivery due to its stability, biocompatibility, biodegradability, sustainability and controlled drug release properties. Casein modified with the natural polysaccharide alginate has also been used to deliver DOX. The self-assembling property of casein in an aqueous medium containing calcium ions resulted in the generation of alginate-coated casein nanocarriers. The calcium ions are responsible for cross linkage between casein and alginate molecules. DOX encapsulated nanocarriers Alg-CasNPs-DOX improved the effectiveness of DOX against the Ehrlich tumor for controlled drug release at the targeted site under acidic conditions in comparison to free DOX [76].

Paclitaxel (PTX), called Taxol in the formulation, is used in chemotherapeutic cancer treatment. It also affects normal cells and has many adverse effects, such as hair loss, low blood pressure, vomiting, nausea, and hypersensitive reactions. A human serum albumin based nanocarrier “Abraxane” shows fewer side effects than Taxol, but its clinical applications are limited due to its high cost. However, casein-based micelles are used for oral delivery of PTX in chemotherapy because it is less expensive and easily degraded by trypsin and cathepsin B (proteolytic enzymes), which are overexpressed by tumors [77]. PTX-loaded sodium caseinate nano micelles (NaCN) have been prepared by the self-assembled property of casein. PTX-loaded micelles showed enhanced tumor accumulation and cytotoxicity against human breast cancer cell line MDA-MB 231 and MCF-7. NaCN is also responsible for the sustained release of PTX at pH 5 and 7.4. Resveratrol or flutamide-loaded casein micelles exhibited similar results [78].

Beta-casein (bCN) micelles are used as effective carriers of antiretroviral formulations that are involved the treatment of HIV infection. Antiretroviral (ARV) formulations encapsulated in bCN in the form of two in one (TRP: EFV) or three-in-one (DRV: EFV: RTV) combinations. The encapsulation of ARV drugs in bCN enhanced the stability and solubility of formulation due to the development of strong interactions between a hydrophobic part of micelles and the drug. The drug-loaded micelles were further encapsulated within microparticles of Eudragit L100 (a polyanionic random copolymer) to protect them from degradation under gastric pH conditions and from enzymatic degradation. The resultant drug carriers showed enhanced bioavailability and oral absorption of ARV drugs [79].

Casein calcium ferrite nanohybrid has been synthesized by desolvation followed by an ionic gelation technique. This nanohybrid conjugated with progesterone ligand has been used to deliver hesperidin. Hesperidin is a bioflavonoid which exhibits antitumor and antioxidant properties. The conjugation of a nanocarrier with progesterone inhibits cancer cell proliferation and targeted drug delivery at the cancer site. The cytotoxicity of hesperidin-loaded nanocarriers was examined against breast cancer cell line MDA-MB-231 and ovarian cancer cell line SKOV-3, resulting in the reduction of LC50 value by 30-fold and drug release by magnetic field stimuli [80,81].

Cinnamaldehyde isolated from *Cinnamomum zeylanicum* shows anti-oxidant, antimicrobial, anti-pyretic and anti-proliferative properties. Casein-calcium ferrite hybrid conjugated with biotin has been used in the delivery of cinnamaldehyde for the treatment of lung tumors. Calcium ferrite nanoparticles are superparamagnetic and are responsible for magnetic field responsive drug delivery. The conjugation of nanocarriers with biotin resulted in the active uptake of carriers by receptors. These drug delivery systems showed pH-sensitive, fast drug release under acidic conditions, in the presence of a magnetic field. The effectivity was examined against L929 fibroblast and A549 lung cancer cells and showed 18-fold reduced LC50 value [82].

Mequindox is an effective antibacterial agent, but its clinical trials have been restricted due to low oral bioavailability. The binding of mequindox with casein improved the bioavailability and solubility of this antibacterial agent. Casein has been considered a good candidate for the delivery of mequindox because it increased the bioavailability of mequindox by 1.20 times and showed the complete release of the drug at the site of infection without reducing efficiency [83].

### 5.4. Silk

Silk is one of the naturally occurring protein polymers that is obtained from the larvae of spiders and silkworm. Silk consists of linear fibrin (a nuclear protein) and serein (adhesive protein) encapsulating the nucleus. Silk is involved in drug delivery due to stability, self-assembly ability, low decomposition rate and a relatively decreased inflammation response at degradation site [26].

eADF4(C16) is a recombinant silk protein obtained from European spiders loaded with positively charged molecules and shows constant drug release at physiological conditions and enhanced drug release under an acidic environment. eADF4(k16) is a polycationic variant of eADF4(C16) that specifically binds with HeLa cells and can carry negatively charged molecules. Different adhesive sequences have also been added into eADF4(C16) proteins to enhance the cell adhesive property of protein. Drugs loaded in the variants of eADF4(C16) showed stimuli responsive release. For example, pH responsive drug carriers are obtained containing a hydroxyl group of protein modified with hydrazine linkers and para-dimethylaminobenzaldehyde [84].

Fibroin is a semi-crystalline structure that compromises 65 to 85% of silk fiber. Fibroin consists of two chains, the heavy chain and the light chain. The heavy chain contains hydrophobic and hydrophilic portion with specific repetitive sequence Gly-X (X = Ala, Ser, Val, Thr, Tyr) in the hydrophobic portion. Fibroin-based drug carriers show better treatment efficiency, stability, solubility with decreased toxicity and drug degradation [43]. Silk fibroin has been conjugated with cRGDfk and Chlorin e6 for the encapsulation of fluorouracil (5-FU), which is involved in the active targeted treatment of gastric cancer. PTX-encapsulated silk fibroin has an antitumor effect against gastric carcinoma [85]. Xie et al. developed curcumin-encapsulated and 5-FU-encapsulated silk fibroin for the inhibition of the colorectal cancer (CRC) cell, which showed more improved activity than free curcumin against cancer cells and had no harmful effect on healthy mucosal epithelial colon cells [86].

Folic acid (FA)-conjugated silk fibroin nanoparticles (SFNP) have been grafted with DOX and, during this this process, were encapsulated into these particles. This double drug loading strategy enhanced the drug loading capacity of nanocarriers. These double loaded FA-SFNPs-DOX-DOX carriers are specifically observed by cervical cancer cells (HeLa cell) and exhibit high cytotoxicity against cancer cells compared to SFPs-DOX-DOX. Cisplatin is an effective drug against many cancers, such as lung, bladder, head and neck, ovarian and testicular cancer. Cisplatin-loaded SFNPs have shown enhanced cellular uptake by lung cancer cells A-549. The conjugation of cisplatin-loaded SFNPs with genipin showed enhanced drug release and high cytotoxicity against cancer cells. Floxuridine (FUDR) is an important hydrophilic anticancer drug used to treat colon and colorectal cancer. FUDR-loaded SFNPs are generated by the nanoparticle self-assembly and show enhanced cellular uptake by HeLa cancer cells and kill 80% of cancer cells. Gemcitabine (Gem) is an anticancer drug used to treat pancreas, bladder and non-small cell carcinoma (NSCLC). Gem-loaded SFNPs are generated by the desolvation process and conjugated with SP5-52 peptide for specific targeting of NSCLC cells [87].

Silk sericin is another essential protein in silk that is water soluble. Sericin exists mainly in an amorphous random coil and sometimes in the form of beta-sheets [44]. Sericin (SER) primarily consists of serine, glycine, aspartic acid, and threonine amino acids. Sericin has many biological properties such as antimicrobial, antioxidant, anti-inflammatory, and anticancer activity due to which it is used in the treatment and diagnosis of diseases.

Sericin blended with Pluronic (F-12 and F-87) has been used for the encapsulation of hydrophobic drugs (PTX) and hydrophilic drugs (FITC-insulin). PTX-loaded nanocarriers are involved in the treatment of breast cancer because these nanoparticles show improved cytotoxic effects against cancer cells (MCF-7) compared to free drugs. SER-Pluronic F-68 nanoparticles loaded with PTX exhibit cytotoxic effect against breast cancer cells. SER-Pluronic F-68 loaded with resveratrol exhibits a prominent cytotoxic effect against colon tumor cells by the accumulation of nanoparticles in cancer cells due to their EPR effect. SER-conjugated with silver engineered nanoparticles (SCS-ENPs) shows stability at different temperatures and pH.

SCS-ENPs have been used in the production of antimicrobial formulations because of their antibacterial activity against *Escherichia coli*, *Staphylococcus aureus* and *Klebsiella pneumoniae*. These nanoparticles have been used for the treatment of sexually transmitted diseases. SER-based charged reversal nanoparticles have been produced by Hu et al. and a cross linking method has been reported consisting of two steps that involve chemical reaction of sericin with chitosan and crosslinking by chemical. The resultant nanoparticles improved the cellular uptake of DOX-loaded nanoparticles. These nanoparticles undergo pH responsive charged reversal. For example, in neutral pH, particles become negatively charged, and in acidic pH, particles become positively charged. Jahanshahi et al. produced sericin-based fluorinated graphene oxide that is used for pH responsive control release of curcumin and has high drug loading capacity. These nanoparticles play an active part in treating a variety of cancers by encouraging apoptosis in SkBr3 human breast/mammary cancer cells, PC-3 prostate cancer cells and HeLa cervical cancer cells [88].

Sericin conjugated with polyethylene glycol and poloxamer nanoparticles is also being used in drug delivery applications [86]. The conjugation of hydrophobic polylactide (PLA) with hydrophilic silk sericin (SS) by using a bis-aryl hydrazone linker resulted in the generation of amphiphilic substance. PLA was modified with terephthalaldehydic acid for the development of aromatic aldehyde terminated PLA (PLA-CHO), and sericin was modified with succinimidyl-6-hydrazino-niccotinamide (S-HyNic). Both modified molecules are mixed in buffer-DMF solution to generate an amphiphilic protein polymer. These amphiphilic molecules self-assembled in water to produce micelles. DOX-encapsulated SS-PLA micelles showed high cytotoxicity for liver cancer cells HepG2.

### 5.5. Elastin

Elastin is present predominantly in the extracellular matrix of arterial walls. It is responsible for the elasticity and flexibility of arteries in body tissues when blood pressure changes. Elastin is in the form of water-soluble topo elastin in nature. These water-soluble precursors are cross linked by covalent bond to form elastin. Elastin-like polymers are developed by genetic engineering technique to obtain desirable properties. Elastin-like polymers are structurally similar to natural elastic, which enables them to escape from the immune system and be used to carry drugs at a specific site in the body [26].

Elastin-like polymers (ELPs) are artificial polypeptides produced by the synthetic genes expressed in *E. coli*, yeast and plants. ELPs exhibit specific hydrophobic pentapeptide motifs Val-Pro-Gly-X-Gly (X is any amino acid except proline). ELPs are soluble at the temperature below characteristic cloud point temperature (Tt) and self-assemble at the temperature above Tt. These are involved in synthesizing diblock copolymers due to their stimulus responsive property [89]. ELPs are biocompatible and exhibit stimulus sensitive responses in the biological environment. Their biodegradation results in the generation of peptides and amino acids that do not adversely affect the body. ELPs are used for the delivery of therapeutics, drugs and radionuclides that are involved in the treatment of cancer, neuroinflammation, type II diabetes and osteoarthritis. Lysine and cysteine in ELPs are reactive sites to bind chemotherapeutic DOX and the incorporation of cleavable linkers in ELPs release the drugs inside the cell [90].

The conjugation of ELPs with DOX results in the generation of micellar structure, which consists of hydrophilic ELPs and hydrophobic drug domain with improved plasma circulation and tumor cell accumulation [91]. Conjugation of radioisotopes with ELPs results in the generation of radionucleotide-conjugated depots used for brachytherapy (a method of treating cancer by irradiating the solid tumor from inside-out). ELPs used in brachytherapy were developed by incorporating I-131 and I-125 into C-terminal at the tyrosine residue of the polypeptide. These depots effectively treat prostate and pancreatic cancer by minimizing exposure to healthy tissues and maximizing radioactive dose delivery to the tumor site.

ELP depots are used for the treatment of diabetes type II by the delivery of glucagon-like peptide-1 (GLP-1), an incretin peptide which controls the release of insulin from pancreatic beta-cells. GLP-1 is released from ELP by injecting protease operated depot (POD) into the surrounding environment. GLP-1 POD in the form of single injection controls blood sugar levels for 5 days. ELP fusion with FGF-21 (fibroblast growth factor 21) is also involved in the treatment of type II diabetes by controlling blood glucose for 5 days. Fusion of cell penetrating peptides (CPP) such as penetration with ELPs improves their drug delivering capacity and cellular uptake and enhances the efficiency of anticancer therapeutics. Modification of ELPs with zwitterion and albumin enhances the drug delivery property. Albumin enhances the circulation time of ELPs in the body by avoiding the interaction of other serum proteins with the surface of ELPs. The modification of the ELP sequence has developed a new class of polypeptide-zwitterionic polypeptides (ZIPPs) to incorporate cationic (lysine) and ionic (glutamic acid) residues to improve the in vivo efficiency of ELP micelles. The incorporation of cationic and ionic residues in the sequence (VPX1X2G) generated stable micelles by the attachment of chemotherapeutic PTX [92].

Lact-ELP fusions have been used for the treatment of dry eye disease (a vision-disturbing and tear-producing chronic ocular surface disease of eyes). The tear-producing protein lacritin (lact) produces tears by stimulating lacrimal glands. Patients with this disease require continual hydrating agents to avoid low lact output. MacKay and coworkers reported the treatment of autoimmune disease and cancer with the aid of therapeutic agent rapamycin and diblock ELPs, respectively. FKBP12 is a binding protein that fuses with the hydrophilic domain of ELPs, and it specifically binds rapamycin on the micellar surface. These nanoparticles, upon administration, show less off-target toxicity, reduce tumor volume and extend circulation time. These rapamycin-encapsulating ELPs are used to treat Sjogren syndrome (an autoimmune disease with endocrine gland inflammation and lymphocytic infiltration) by reducing lymphocytic infiltration in the lacrimal gland and reducing the inflammation of glands [93].

Single-stranded DNA (ssDNA) has been enzymatically conjugated with ELPs with the help of the catalytic domain of Porcine Circovirus type-2 replication initiator protein (pRep). ELPs first fused with pRep and then covalently bounded with 5′ phosphate of cleaves ssDNA. This DNA-displaying nanoparticle encapsulates PTX and is conjugated with DNA aptamer, which specifically binds with Mucin-1 (MUC1) protein overexpressed by cancer cells. The nanoparticles upon interaction with MUC1 release PTX and induce cancer cell death [94].

Silk-elastin-like proteins (SELPs) consist of a block from silk (GAGAGS) that provides the thermal and chemical stability, mechanical tunability and crosslinking sites and a block of topo elastin (GVGVP) that provides dynamic functions on exposure to different environmental stimuli by undergoing reversible structural transitions. A SELP fusion system has been used to deliver endothelial growth factors involved in treating kidney diseases [95].

### 5.6. Zein

Zein, a well-known plant protein, is usually derived from corn and maize. The main components of zein include non-polar amino acids such as glutamic acid, proline, leucine and alanine, which are responsible for zein proteins’ hydrophobic character. Solubility in water can be improved by the addition of alcohol, urea, alkali or ionic detergent [44]. Zein is used in the manufacturing of different products such as cloths, waterproof papers, food products and pharmaceutical products. FDA approves it as a safe excipient of drugs. It is used in manufacturing oral formulations due to its controlled release properties. It is used as a coating material due to its film-forming and fiber-forming properties [89].

Zein synthesized by desolvation method and coated with PEG with mucus permeating property has been used for oral drug delivery. Mucus permeating nanocarriers minimize interaction with mucus mesh increase the mobility of nanocarriers and enhance diffusion through the protective layer. The coating of PEG on zein decreased zein’s hydrophobicity and increased the nanocarrier’s hydrophilicity, enhancing the mobility of nanocarriers in the intestinal mucus. After oral administration, PEG-coated zein nanoparticles were entrapped in mucus mesh; then PEG was released and crossed the protective mucus layer to reach epithelium [96]. Curcumin encapsulated into zein exhibited 9-fold increased oral bioavailability compared to commercially available curcumin [95]. Curcumin isolated from Curcumalonga exhibits antioxidant, anticarcinogenic and anti-inflammatory properties, but clinical trials are limited due to poor solubility and rapid degradation by metabolism.

Nanocarriers that are based on animated mesoporous silica nanoparticles (AMSNs) and zein has been used for the delivery of 5-Fluorouracil (5-FU) and curcumin (CUR). AMSNs are efficient drug carriers due to their large surface area, uniform pore size, stability, biocompatibility and high pore volume. AMSN can be modified by the condensation process and is responsible for drug release in response to different stimuli such as temperature, pH, light, enzymes and redox reaction. 5-FU is an analogue of pyrimidine that exhibits antimicrobial, antineoplastic properties and is effective against cancer. AMSN prepared by condensation process has been used to carry CUR inside its pores. Zein conjugated with glycyrrhetinic acid has been used to carry 5-FU and acted as a gatekeeper for AMSN. The resulting nanocarriers served as efficient carriers for anticancer drugs and showed high toxicity and pH responsive drug release at pH 7.4 and 5.5 [97].

Mucus permeating poly(anhydride)-thiamine (GT) coated zein displayed that the particle size of 250 nm has been used for insulin delivery. These nanocarriers’ oral absorption and bioavailability were investigated in *C. elegans* and diabetic Wistar rat models. The GT-coated zein nanoparticles improved the intestinal absorption and bioavailability of insulin through the oral route of administration as compared to conventional insulin solution. The resulting nanoparticles reduced blood glucose levels by up to 20% and reduced fat accumulation in the body [96,98].

Honokiol (HNK) is a biphenolic compound used to treat various tumors such as the brain, colon, liver, breast, lungs and skin. Zein conjugated with hyaluronic acid (HA) encapsulated HNK has been used to target HNK in breast cancer therapy. The obtained nanoparticles HA-Zein-HNK, having a size of 210.4 nm, showed improved antiproliferative and apoptotic activity against cancer cells 4TI in mice. HA-Zein-HNK exhibited efficient antitumor activity by suppressing the Vimentin expression and regulating the E-cadherin expression [99]. Zein modified with Poly(ethylene) oxide (PEO) has been used in the chemotherapy of human gallbladder cancer by the entrapment of Gallic acid (GA). GA loaded inside nanocarriers exhibited high cytotoxicity against cancer cells and are responsible for the controlled release of GA at tumor site [100].

Sodium caseinate (S-CAS) stabilized zein nanoparticles and sodium carboxymethyl cellulose (S-CMC) stabilized zein nanoparticles have been used for loading PTX and 10-hydroxycamptothecin (HCPT), which exhibited enhanced cytotoxicity against tumor cells, more retention time and sustained drug release as compared to free drugs [101]. Zein has also been used to develop a multidrug carrier that delivers a third-generation aromatase inhibitor, exemestane (EXM), in the treatment of hormone-dependent breast cancer. Multidrug carriers based on zein can encapsulate both Bortezomib (proteasomal inhibitor) and Vorinostate (histone deacetylase inhibitor) that cause cancer cell death by apoptosis. The zein-based multidrug carriers prepared from zein, poloxamer, and lecithin showed pH sensitive release of active substances under acidic conditions [102].

### 5.7. Gliadin

Gliadin is present in wheat gluten, a complex of proteins (glutenin and gliadin) and carbohydrates. Nearly 40% of gliadin consists of amino acid glutamine and proline. Gliadin is slightly soluble in an aqueous solution, similar to creatine. Gliadin is present in skin formulation because of its interaction with skin creatine. It is involved in a controlled drug release system and carries hydrophobic and amphiphilic compounds such as amoxicillin, vitamin A and vitamin E. Gliadin is also used in preparing oral formulations because gliadin can bind with mucosa by hydrogen bonding and with the cell membrane by hydrophobic interactions. It is effective in the treatment of gastric ulcers because it removes *Helicobacter pylori* from the mucosa of the organ [26].

Gliadin has been used in the oral formulation and provides site-specific release of active substances. The conjugation of gliadin with Dolichos biflorus lectin (DBA) improved the adhesive property of nanocarriers in the colon due to the presence of the N-acetyl-D-galactosamine group. It reduced the interaction of nanocarriers with duodenum and jejunum mucosa. Moreover, the conjugation of gliadin with Ulex europeus lectin (UE) enhanced the interaction between nanocarriers and bovine submaxillary gland mucin. Gliadin has been used to treat diseases related to *Helicobacter pylori* (*H. pylori*), a well-known bacterium responsible for intestinal and gastric disorders. Gliadin has been used as a carrier for antibiotics such as clarithromycin and amoxicillin that are used for the treatment of *H. pylori*-related diseases. The conjugation of gliadin with UE and Concanavalin A-lectin (Con-A) encapsulate acetohydroxamine (AHA) that inhibits the production of the Urease enzyme used by bacteria for proliferation. Amoxicillin encapsulated gliadin nanoparticles have been used for the treatment of bacterial infections by inhibiting bacterial growth within 8 to 12 h of drug administration. Gliadin nanoparticles exhibited controlled release of antibiotics due to the strong interaction of gliadin with gastric mucosa and high retention rate. Gliadin loaded with antibiotic arithromycin and protein pump inhibitor PPI (omeprazole) inhibits the growth of *H. pylori* bacteria. Gliadin-based nanoparticles have been used for the development of structures consisting of tetanus toxoid, which is effective against tetanus. The conjugation of gliadin with chitosan stabilizes the drug and is responsible for controlled drug release in response to pH stimuli. Meletin (quercetin) is also entrapped into gliadin into matrix-like structures [103].

The conjugation of gliadin with gelatin generates nanocarriers that encapsulate the anticancer drug cyclophosphamide. Cyclophosphamide encapsulated in gliadin nanoparticles has been used in the treatment of breast cancer and showed high cytotoxicity against MCF-7 cancer cells compared to free cyclophosphamide due to the controlled release of the drug at the targeted site [101]. PTX-loaded zein nanoparticles were obtained by desolvation and film hydration and stabilized by Pluronic 127 (P127). The P127 stabilized zein nanoparticles showed decreased size due to favorable interaction between zein and P127. The effectivity of PTX-loaded nanocarriers was assessed on cancer cells of MCF-7 and MDA-MB-231, which showed higher LC50 values than free PTX. All-trans retinoic acid (ATRA), a lipophilic compound that is a metabolite of vitamin-A loaded inside gliadin for the treatment of various cancers because of antitumor property of ATRA. ATRA-loaded gliadin nanoparticles generated by the desolvation technique were found effective against actinic keratosis (pancreatic lesions) and acute promyelocytic leukemia. Curcumin loaded inside lectin conjugated gliadin prepared by desolvation technique exhibited enhanced antioxidant, antitumor and wound-healing properties. Folate-conjugate gliadin nanoparticles exhibited a controlled release of curcumin compared to pure gliadin, which exhibited a burst release of the drug. Folate is also responsible for the selective uptake of nanocarriers by the cells expressing folate receptors [102].

### 5.8. Soy Proteins

Soy proteins are plant proteins which are a combination of polar and non-polar amino acids. The significant components of soy protein isolate (SPI) are glycinin and beta-conglycinin. In an aqueous medium, soy proteins consist of a hydrophobic nucleus and a hydrophilic shell. SPI undergoes desolation and coacervation to give drug-releasing soy protein nanoparticles [26]. Soy proteins are used as nanocarriers for delivering hydrophobic drugs and nutraceuticals to the hydrophobic nature of the surface. Beta-conglycinin is used primarily for nanocarriers because it has a simple structure and is easy to prepare compared to glycinin [104].

The conjugation of soy protein with fucoidan generated core–shell structured nanoparticles that were used to encapsulate curcumin and other lipid soluble drugs through electrostatic interactions. Fucoidan is a polysaccharide found in the cell wall of algae which contains L-fructose, a sulfur ester group, aldehydes, mannose, xylose and glucuronic acid. Curcumin was distributed inside SPI matrix evenly and showed better dispensability and stability during storage. The nanocarriers having a size of 236.56 nm and drug loading capacity of 95% are responsible for the targeted delivery of curcumin in the intestine [105].

Soy protein nanoparticles prepared by desolvation of ethanol and stabilized by glutaraldehyde (cross-linker) have been used for carrying curcumin. The curcumin-loaded soy protein nanoparticles have a diameter of 220 and 287 nm with 97% drug loading efficiency. These nanocarriers are responsible for the sustained release of the drug; about 80% of the drug is released within 8 h of drug uptake. Cellulose conjugated soy protein nanocarriers having a diameter of 50–52 nm and drug loading efficiency of 88% are considered efficient curcumin carriers. The resultant nanocarriers exhibited improved targeted drug delivery, thermal stability, and loading efficiency of nanocarriers retained even after freeze drying. Folic acid conjugated soy protein nanocarriers for encapsulating curcumin exhibit a faster drug release rate and 92.7% drug loading efficiency. Conjugation of soy protein with beta-conglycinin, a storage globulin, generated core–shell-structured nanoparticles for the delivery of curcumin. The resultant nanoparticles exhibited improved dispersion, stability, bioavailability, cytotoxicity and sustained drug release of about 56–60% within 24 h. Soy protein conjugated with IgG (snake antivenom) in the presence of conjugating agent 1-ethyl-3-[3-dimethylaminopropyl] carbodiimide hydrochloride (EDC). The encapsulated antivenom nanoparticles inhibit the activity of enzymes such as protease, phospholipase and hyaluronidase produced by Bungarus caeruleus venom. Soy protein nanoparticles conjugated with folic acid and cross-linked with 1-ethyl-3-[3-dimethylaminopropyl] carbodiimide hydrochloride (EDC) and N-hydroxysuccinamide (NHS) have been used for encapsulating DOX. DOX-loaded nanocarriers showed a loading efficiency of 23%, improved accumulation, penetration and cytotoxicity against tumor cells. phenylboronic acid modified nanoparticles have been used for the delivery of anticancer drugs such as DOX. The modified nanocarriers decrease tumor interstitial fluid pressure and show a high affinity for sialic acid in tumor cells [106].

Encapsulation of docetaxel in soy protein nanoparticles increased the size of nanoparticles and exerted high cellular uptake by A549 tumor cells. Docetaxel-loaded soy protein exhibited efficient accumulation at the tumor site, lower LC50 value and improved apoptotic activity compared to free drug [102].

### 5.9. Albumin

Albumin is an important protein present in blood plasma and has thiol, carboxyl and amine groups, due to which it is easy to modify its surface. It is a flexible protein owing to the disulfide bond; structure can be easily modified under mild conditions and easily return to its original form. It is obtained from egg white (ovalbumin), BSA, human serum albumin, milk, soy and legumes. It is easily absorbed by inflamed tumor tissues and involved in the treatment of shock, burns, respiratory problems, trauma, blood dialysis and cardiopulmonary surgery—see Field [26]. Albumin has a high concentration of aspartic acid, glutamic acid, lysine, cysteine and arginine. Albumin is used as a carrier for ocular drug administration because the retention time of albumin is higher in inflamed eyes than in healthy tissues due to its EPR effect—see Field [106]. Albumin-based drug carriers involved in treating diabetes, hepatitis, arthritis, cancer and viral disease are commercially available.

PEG-modified albumin nanoparticles have enhanced blood circulation time and improved nanoparticle movement across the respiratory tract’s mucus layer. Albumin nanoparticles are involved in the delivery of DOX, Abraxane, curcumin and tacrolimus involved in cancer treatment. Kim et al. generated curcumin-loaded human serum albumin (HAS) that showed high solubility, and Dries et al. developed DOX-loaded HSA nanoparticles to reduce the side effects of anticancer drugs. Iwao et al. developed HSA and myeloperoxidase (MPO) nanoparticles conjugated with 5-aminosalicylic acid (5-ASA) for the treatment of ulcerative colitis [107].

Aljabali et al. prepared piceatannol-loaded BSA that improved the solubility, bioavailability and anticancer activity of piceatannol that is involved in the treatment of colon cancer. These nanoparticles target colon cancer cells (CaCo-2 and HT-29 cells) and suppress the growth of tumor cells. Jithan et al. prepared curcumin-loaded BSA nanoparticles that are involved in the treatment of breast cancer. These nanoparticles target breast cancer cells (MDA-MB-231 cells) and exhibit enhanced antitumor activity and sustained release of curcumin for the treatment of cancer. Lee et al. developed paclitaxel-loaded PEGylated albumin nanoparticles that exhibit the improved activity of paclitaxel against cancer cells. paclitaxel-loaded PEGylated albumin nanoparticles have increased circulation time and improved anticancer activity. Abbasi et al. prepared positive charge carrying DOX-loaded albumin nanoparticles for breast cancer treatment. Polyethyleneimine induced positive charge stability to nanoparticles and improved the cellular uptake of the drug by MCF-7 cells [47].

A variety of ligands have been used for the modification of nanocarriers for targeted drug delivery. Hyaluronic acid (HA), a natural glycosaminoglycan is a commonly used ligand that binds receptors such as clusters of differentiation-44 (CD44) that are over-expressed by tumor cells and involved in drug delivery for tumor, tissue engineering and joint diseases. HA-modified albumin nanocarriers showed enhanced biocompatibility, stability, pH-responsive targeted drug delivery and improved hydrophilicity of nanoparticles. HA-modified nanocarriers show interaction with CD44-like receptors also expressed by some tumor cells [108].

Cisplatin-loaded bovine albumin nanoparticles (CPT-BSANPs) prepared by the desolvation technique exhibited improved anticancer activity compared to free cisplatin when tested on the cell line MCF-7. MCF-7 cells are divided into two groups beta-cyclodextrin treated and beta-cyclodextrin untreated, beta-cyclodextrin cells exhibited increased cellular uptake and apoptosis upon CPT-BSANPs introduction [109].

The technique of modifying protein with a site-specific polymer chain conjugated in biotin has been used to generate polymers directly from proteins such as BSA. Protein-initiated atom transfer radical polymerization (ATRP) has been utilized to obtain amphiphilic molecules that can self-assemble. Maleimide-modified ATRP initiators functionalize the thiol group on BSA. Such nanoaggregates have been used to deliver enzymes without affecting their catalytic activity and eliminate organic solvent use during their synthesis [110].

Modification of human serum albumin (HAS) with P-selectin targeted peptide (PSA) and IR780 resulted in the development of nanocarriers that act as photosensitizers. HAS-modified with a photothermal agent (IR780) and PSA peptide specifically binds with P-selectin, which is overexpressed on platelets. The photosensitive PSN-modified albumin-based nanocarriers are used to encapsulate PTX and produce PSN-HAS-PTX-IR780. The albumin-based nanocarrier PSN-HAS-PTX-IR780 showed enhanced drug delivery at the tumor site under mild temperature conditions, improved drug accumulation that enhanced therapeutic activity against the 4TI primary tumor and inhibited the metastasis by binding with metastasis-infiltrating platelets (platelet bridge) [111].

### 5.10. Keratin

Keratin is a structural fibrous protein in wool, feathers, beak, hair, nails, hooves, claws, horns and the outer skin layer, thus protecting the body [112]. The keratin structure is hydrophobic, and the disulfide bond in the keratin structure provides resistance to the chemical and enzymatic environment. The presence of hydrophobic interaction, hydrogen and ionic bonds between amino acids, provides stability to keratin. Keratin is involved in drug delivery due to its abundance in nature, intrinsic biocompatibility and mechanical durability [44]. Keratin-based drug carriers are responsible for the targeted delivery of drugs for treatment of cancer by binding to specific vitronectin integrin receptors that are highly expressed by cancer cells due to the specific sequence of amino acids arginine-glycine-aspartic acid (RGD) and leucine-aspartic acid-valine (LDV) [112].

Dual stimuli-responsive keratin nanoparticles were developed by Li et al. for the delivery of DOX at targeted site. Keratin-coated DOX nanoparticles (K-DOX-NPs) accumulate in the tumor site and demonstrate pH and redox-responsive aspects to release the drug at targeted site. K-DOX-NPs are biocompatible and exhibit high toxicity to human lung carcinogenic cells A549 [113].

DOX-loaded keratin nanoparticles were developed by the electrostatic interaction between negatively charged keratin and DOX, which is used as a drug carrier for cancer treatment by Aluigi’s group. Drugs are loaded into these nanocarriers by hydrophobic interaction between the hydrophobic part made of DOX and the hydrophilic part keratin. The stability of DOX-loaded nanoparticles increased by blending protein with hyaluronic acid, which reduced particle size up to 50–100 nm and gave pH and reduction sensitivity to nanocarriers. The stability of nanoparticles was further increased by introducing hydrogen peroxide interaction with the sulfhydryl group of keratin, which made it responsive to reduction and exhibited high cytotoxicity against cancer cells compared to free DOX. The modification of keratin with catechins (oligomer) through formaldehyde in an aqueous medium resulted in the generation of drug carriers that deliver the drug through physical adsorption on the nanocarriers and enhance drug stability in aqueous medium for a long time. The reaction between the sulfhydryl group on keratin and the double bond of polymer generated copolymers K-g-PHPMA and K-g-PEG with the keratin-rich core. Such DOX-loaded micelles showed drug release in response to trypsin. The conjugation of Pluronic (temperature-sensitive polymer) with keratin (reduction-sensitive polymer) generated copolymer, which has been used to encapsulate curcumin and showed control drug release under reduction conditions and in the presence of trypsin [114].

Mesoporous silica nanoparticles (MSNs) have been used in pharmaceutical applications due to high biostability, biocompatibility, large surface area and high drug loading efficiency. However, its use for the clinical trial is limited due to its cytotoxicity against normal cells and premature drug release. Conjugation of polydopamine-coated MSH with keratin through iron (III) mediated coordinate formation has been used to load DOX, and resultant nanocarriers exhibited pH and GSH responsive drug release and low cytotoxicity against normal body cells [115].

Keratin modified with poly (ethylene glycol) through disulfide linkage and conjugated with DOX has been used to design prodrug PK-SS-D for reducing response targeted drug delivery at the tumor site. This protein-based prodrug self-assembled to form micelles with a diameter of 175 nm and drug loading efficiency of 20%. Approximately 52% of drugs were released at the tumor site with less drug leakage of about 17% within 10 days, but exhibited less antitumor activity than free DOX [116]. Keratin extracted from human hair conjugated with poly (2-methacryloxyethyl phosphatidylcholine) (MPC) generated micelles KPC have encapsulated DOX. These micelles prepared by the thiol chain transfer radical polymerization process exhibited pH, enzyme and GSH triple responsive targeted drug delivery at the target site and enhanced cytotoxicity against A549 and HEK-293 tumor cells. The DOX-loaded KPC micelles showed better stability, prolonged circulation time and improved therapeutic efficiency than free DOX [117].

The efficiency of photodynamic therapy in cancer treatment is limited due to limited oxygen levels at the tumor site, non-targeted delivery of photosensitizer and low production of nitric oxide, which has a vital role in cancer treatment because it can suppress tumor cell growth, enhance apoptotic mechanism and reverse multidrug resistance. Modification of keratin (NO donor) with targeted ligand phenyl boronic acid (PBA) and photosensitizer methylene blue (MB) improved photodynamic therapy (PDT) for tumor treatment. Further modification of these nanocarriers with D-alpha-tocopherol polyethylene glycol 1000 succinate specifically suppresses breast cancer cells’ growth. The resulting nanoparticles showed enhanced cellular uptake by 4TI breast cancer cells in mice. Moreover, these nanoparticles improved the efficiency of photodynamic therapy by reducing the amount of glutathione and increasing NO production intracellularly [118].

## 6. Challenges in Advancing Protein Derived Drug Delivery Carriers

Protein-based carriers are used for the delivery of anticancer drugs, DNA, RNA, hormone and growth factors due to their biocompatibility, biodegradability and cost-effectiveness. However, only a few of them have been approved for clinical trials due to the different complexities of drug carriers and the need for regulatory guidelines.

- The use of protein-based carriers requires the modification of protein structure that may result in the loss of native properties of protein and loss of activity. If endotoxin is attached to protein or the transmission of prions is required, a low yield of protein-based nanoparticles is obtained, and rapid degradation of carriers takes place [44].
- Mostly, protein-based drug delivery carriers cannot release drugs for an extended period because proteins are hydrophilic, and their nanocarriers swell on absorbing water inside the body and release drugs rapidly. The use of chemical linkers to stabilize their structure for a long time is often toxic.
- When animal protein sources are used in the development of drug carriers, there is a possibility of transferring animal diseases to humans [26].
- Drug-loaded polymeric micelles for clinical trials depend on passive targeting and enhanced permeability and retention (EPR) effect. Their targeting effect is less clear and pronounced than in animals and single cells due to the complexity of the human body and tumor.
- Many stimuli-responsive polymeric micelles have been generated, but a few of them have entered the clinical stage due to these challenges related to their use as drug carriers [119]. The distribution of internal stimuli is not specified, which may result in off-target drug release due to insufficient sensitivity to stimuli. For example, some normal tissues can find overexpression of enzymes, low pH and high concentration of GSH.
- Changes in the manufacturing process of protein-based drug carriers change the physiochemical properties of proteins, affecting the efficiency of drug carriers. The manufacturing process also involves drug loading in polymeric micelles. For example, for efficient drug loading in the dialysis process, 36 h are required, and the chlorinated solvent is used in the emulsification method that is not safe [119].
- The modification of structure affects the stability of micelles. For example, micelles are not disassociated in water at a concentration above critical micelle concentration (CMC), but when exposed to serum proteins, most micelles disassociate and bind to serum protein. These complexities limit large-scale production and clinical trials of drug-loaded micelles [120].
- Other barriers relevant to using nanocarriers at a large scale include the diversity of cancer. Some types of cancers are not determined yet, and the physical nature of cancers varies from person to person.
- For targeted drug delivery, the surface of nanocarriers is modified with ligands that increase the manufacturing steps, increasing the cost of the product [121].
- Some other obstacles in commercializing nanotherapeutics include the separation of by-products and starting materials from nanocarriers, limited knowledge of the interaction of nanosystems with living cells, lack of funds and reluctance of pharmaceutical industries to invest in the novel nanotherapeutics [122].

## 7. Regulatory Aspects

The major government organization working on the regulation of nanocarriers is the FDA through the Center for Biologics Evaluation and Research (CBER), the Center for Devices and Radiological Health (CDRH) and the Center for Drug Evaluation and Research (CDER). The effect of nanotechnology on the environment and human health behavior of nanomaterial under different environmental conditions should be taken into account during nanotechnology research. The assessment of biological properties of nanocarriers and associated risks, the assessment of instruments and methods of characterization of nanocarriers should be considered [123].

Various regulatory agencies are working to evaluate these formulations’ toxicity, safety and biocompatibility. Different protocols developed for assessing nanomedicine depend on the active principle ingredient (API) entrapped. Therefore, some regulations and guidelines were developed to minimize the risk related to the use of nanomedicines and their carriers. Any formulation containing a substance or drug that triggers the immune system to show a response should have strict regulatory control. The FDA developed regulatory protocols for the assessment of drugs.

- Before starting any conventional or nanomedicine drug clinical trial, the Investigational New Drug (IND) application must be sent to the FDA. The FDA reviews the IND application to assure the safety of patients in the clinical trial.
- The FDA must be notified if any change is made in the ingredients, manufacturing process or quality testing of an existing drug that may affect the properties of the drug. After the FDA approves, the formulation could be used for a clinical trial.
- Nano products have different properties from their bulk counterpart due to their small size, so these are always considered New Molecular Entities (NMEs). The FDA evaluates the risk-benefit ratio of the formulation before approving it for a clinical trial.

According to some experts and critics, the regulatory protocols developed by the FDA are insufficient to determine the safety of nanomedicines and carriers. Due to the need for regulatory guidelines for the approval of nanomedicine, the clinical use of nanomedicines is limited [124].

Clinical use of nanoproducts (nanoparticles, polymeric micelles, liposomes) depends on their characterization, assessments, and proper understanding of their properties. Formulations based on proteins, peptides, and antibodies must follow regulatory guidelines developed for biomedical, medical products and new chemical entities NCEs. During the assessment, the interaction of nano products with immune cells and plasma proteins must be taken into consideration [125].

European regulators have recognized some domains that require further development regarding drug regulation, physiochemical properties that strongly affect the pharmacological activity of drugs and studies related to penetration, absorption, permeation and targeting capacity to test the toxicity of nanomaterial and to test biological and mechanical routes. For the regulation of nanomedicines and their effective use, efforts are being made toward developing OECD (Organization for Economic Co-operation and Development) guidelines, including acute, sub-acute and chronic studies of the toxicity of chemicals. According to researchers and regulators, several regulatory aspects could be revealed by analyzing the life cycle of nanomaterials, including laws related to chemicals, toxicity, waste products and agriculture [126].

Manufacturing nanoproducts is a multi-step process; information related to the quality and control of intermediates obtained during manufacturing has to be identified that helps to understand parameters such as temperature, pH, time, stirring rate, etc., involved in the development of medicines. The compatibility of excipients with active substances should be developed, and the excipient’s properties and concentration that affect the drug performance must be considered. Additional considerations are applied when a new excipient is chosen for drug delivery or a new route of excipient administration is preferred. In addition to toxicological evaluation, data related to identification tests, physical characteristics and purity level are necessary depending on the dosage forms of medicines. For example, nanosuspension nanoparticles require standard pharmacopoeial tests that include size distribution, re-dispersibility, reconstitution time, uniformity of dosage time and rheological properties. The purpose of these tests is to determine the quality of the drug and to investigate the effect of temperature, humidity and light on the drug [125].

## 8. Summary and Future Perspectives

An advanced study in molecular medicine, biochemistry, immunology, and nanomedicines will be helpful for more effective treatment and diagnosis of diseases in the near future. The evolution in the field of nanotechnology and nanomedicine provides a novel and effective alternative to conventional treatments and open up new opportunities for early diagnosis and effective treatment of diseases [126].

Over time, the demand and need for biocompatible protein-based drug carriers is increasing in the medical field. Future research must focus on the large-scale production of these carriers to fulfill the demand for protein-based drug delivery carriers. To provide a suitable drug delivery vehicle and to reduce off-target drug release, the structure and properties of proteins must be changed accordingly [26].

With the emergence of polymeric micelles as drug carriers, most of the focus has been on diblock and triblock copolymers. A study on the usage of tetrablock and pentablock copolymers for drug delivery has been conducted recently [119].

The low yield of protein-based nanoparticles due to structural modification can be overcome using a recombinant protein approach and research on improving downstream protein processing.

The problem of rapid drug release from protein-based carriers can be solved by blending proteins with suitable biocompatible polymers [44].

Although many materials have been developed that can be used as drug carriers, using PEG as a hydrophilic component of micelles is consensus because PEG is FDA-approved. Future research is required to facilitate the translation of new material platforms so that the list of approved materials can be expanded to newly synthesized more efficient medicines [120].

The development of DNA/RNA nanocarriers to remove cancer cells could be a promising research area because anti-cancer therapies based on DNA/RNA are safer and more effective for cancer treatment [122]. Most of the FDA-approved nanocarriers have been used to treat cancer and neurologic disorders for the last three decades. Future research will be on the usage of nanocarriers for microbial infection treatment.

## Data Availability

Not applicable.
